# Identification and evaluation of gut microbiome as non-invasive biomarkers for early lung adenocarcinoma from a multi-center study

**DOI:** 10.3389/fcimb.2026.1813261

**Published:** 2026-05-14

**Authors:** Weidong Zhang, Shurui Wu, Leilei Shen, Peng Peng, Rong Li, Qian Zheng, Xiaodong Jia, Qingyan Liu, Yang Liu

**Affiliations:** 1 Anesthesia and Operation Centre, The Fifth Medical Centre of Chinese PLA General Hospital, Beijing, China; 2 Department of Thoracic Surgery, The First Medical Centre of Chinese PLA General Hospital, Beijing, China; 3 Department of Thoracic Surgery, Hainan Hospital of Chinese PLA General Hospital, Hainan, China; 4 Department of Anesthesiology, The Second Affiliated Hospital of Soochow University, Suzhou, Jiangsu, China; 5 Department of Health Medicine, The Second Medical Centre & National Clinical Research Centre for Geriatric Diseases of Chinese PLA General Hospital, Beijing, China; 6 Department of Oncology, The Fifth Medical Centre of Chinese PLA General Hospital, Beijing, China

**Keywords:** dignostic assessment, gastrointestinal microbiome, lung adenocarcinoma (LUAD) of lung, machine learning (ML), random forest

## Abstract

**Objective:**

This study aims to characterize the GM in LUAD patients and develop and validate a GM-based diagnostic model for LUAD.

**Methods:**

In this prospective, randomized, multi-center study, the GM was characterized, and an LUAD classifier was developed using a training cohort of 175 early-stage LUAD patients and 107 healthy controls. The model was further validated in a test cohort, two independent external cohorts from Jiangsu and Hainan, and an advanced LUAD cohort. Additional ML models were also developed and compared to assess their predictive performance.

**Results:**

LUAD patients exhibited reduced microbial diversity and significantly altered microbial composition compared to healthy controls. The phylum Verrucomicrobia and 13 genera, including *Enterococcus* and *Akkermansia*, were more abundant in the LUAD group, while 5 phyla, such as Fusobacteria and Cyanobacteria, and 17 genera, including *Lactobacillus* and *Weissella*, were enriched in the control group. Using random forest (RF), eight operational taxonomic units were identified as the optimal subset, achieving an area under the curve (AUC) of 0.998 in the training cohort and maintaining high accuracy in the test cohort (AUC = 96.9%). The model also demonstrated robust performance in two independent cohorts from Jiangsu (AUC = 97.6%) and Hainan (AUC = 82.9%), with strong diagnostic potential for advanced LUAD. Among five common models, the RF model exhibited the highest diagnostic accuracy.

**Conclusions:**

This study provides a comprehensive characterization of the gut microbiome in LUAD and develops a diagnostic model based on microbial biomarkers, which is validated across regionally diverse cohorts, highlighting its potential as a reliable and non-invasive screening tool for LUAD.

## Introduction

Lung cancer (LC) remains the leading cause of newly diagnosed cancers and cancer-related deaths globally, accounting for 12.4% of new cases and 18.7% of deaths in 2022 (Bray et al., 2024). Histologically, over 80% of LC cases are classified as non-small cell lung cancer (NSCLC), withLUAD representing more than 50% of these cases and showing a rising incidence in recent years (Laguna et al., 2024). Due to the asymptomatic nature of early-stage LC and the limitations of current diagnostic methods, most patients are diagnosed at advanced stages, resulting in a poor prognosis with a five-year survival rate of 20-30% (Cancer of the lung and bronchus - cancer stat facts; Herbst et al., 2018). Early detection significantly improves survival outcomes (Chen et al., 2024), highlighting the need for efficient and robust diagnostic biomarkers for LUAD.

While smoking is a major risk factor for LC, its association with LUAD is weaker compared to other LC subtypes, such as squamous cell carcinoma and small cell carcinoma, suggesting the involvement of other key risk factors in LUAD onset and progression (Bano et al., 2025; Yiminniyaze et al., 2025). Increasing evidence points to a significant correlation between LC and the gut microbiome (Liu et al., 2019; Qin et al., 2022; Luo et al., 2024; Jin et al., 2025). The human gut microbiome, a critical microbial system, plays a vital role in nutrient extraction, metabolism, immunity, and inflammation (Bouskra et al., 2008; Cho and Blaser, 2012), and is implicated in various diseases, including cancer, diabetes, respiratory conditions, and neurological disorders (de Vos et al., 2022). The gut microbiome is characterized by stability, resilience, and responsiveness to pathological changes, making it a valuable diagnostic target for multiple diseases, including cancer (Eslami et al., 2025). Several large-scale studies have successfully developed diagnostic models based on the GMfor specific cancers, such as colorectal cancer (CRC), hepatocellular carcinoma (HCC), and pancreatic cancer (PC), highlighting the potential of microbial markers as targeted, non-invasive diagnostic tools (Yu et al., 2017; Ren et al., 2019; Nagata et al., 2022). However, the GM signatures and diagnostic potential for LUAD remain largely unexplored.

This study collected 531 fecal samples from regions in North, East, and South China, with 472 samples undergoing 16S rRNA sequencing. A training cohort consisting of 107 healthy controls and 175 early-stage LUAD patients was used to characterize the GM associated with LUAD and develop a gut microbial classifier to differentiate LUAD patients from healthy individuals. The classifier was further validated using a test cohort, as well as two independent cohorts from Jiangsu and Hainan, and an advanced LUAD cohort, to assess the GM’s potential as an auxiliary screening tool for LUAD. To construct a robust and efficient diagnostic model, five distinct ML models were trained and validated using their respective candidate operational taxonomic units (OTUs), and their diagnostic performances were compared.

## Methods

### Study design and participants

This study adhered to the Transparent Reporting of a multivariable prediction model for individual prognosis or diagnosis (TRIPOD) reporting guidelines (Collins et al., 2015) and was approved by the Institutional Review Board of the Chinese PLA General Hospital (S2021-407-01). All participants provided informed consent for the use of their fecal samples and clinical data. Eligible participants were healthy volunteers and early-stage LUAD patients (Tumor stage I or II) aged between 18 and 80 years. Exclusion criteria included individuals with malignant tumors other than LUAD, those who had undergone prior therapies such as surgery, targeted therapy, or chemotherapy for their current LUAD, individuals with gastrointestinal disorders, those who had received antibiotics or probiotics within the last 12 weeks, and those with missing clinical information. All LUAD patients had a confirmed postoperative histopathological diagnosis. Tumor staging was based on the eighth edition of the TNM classification for LC by the International Association for the Study of Lung Cancer (IASLC). The control group consisted of healthy volunteers who were hospitalized for routine physical examinations. Healthy participants underwent a medical history review, physical examination, chest computerized tomography (CT) scan, and laboratory tests to confirm their health status. A total of 531 fecal samples were collected between August 2022 and June 2024 from the Chinese PLA General Hospital (Beijing, China), Hainan Hospital of the Chinese PLA General Hospital (Hainan, China), and the Second Affiliated Hospital of Soochow University (Jiangsu, China). Of these, 472 samples underwent 16S rRNA sequencing. Clinical data for all participants were obtained from their electronic medical records.

### Sample collection, DNA extraction, and polymerase chain reaction amplification

Fecal samples were freshly collected using sterile plastic tubes on ice and stored at -80 °C until processing. DNA was extracted using the HiPure Stool DNA Kit (Magen, China) following the manufacturer’s instructions. DNA concentration and purity were measured with a NanoDrop2000 (Thermo Scientific, USA), and DNA quality was assessed by agarose gel electrophoresis. The V3–V4 region of the 16S rRNA gene was amplified *via* PCR using specific barcoded primers, 341F (5’-CCTACGGGNGGCWGCAG-3’) and 806R (5’-GGACTACHVGGGTATCTAAT-3’). Amplicons were purified using the AxyPrep DNA Gel Extraction Kit (Axygen Biosciences, USA), and their quantity was determined using the ABI StepOnePlus Real-Time PCR System (Life Technologies, USA). Paired-end sequencing (PE250) was performed on the Illumina platform. Raw sequencing data have been deposited in the Genome Sequence Archive at the National Genomics Data Center, China National Center for Bioinformation/Beijing Institute of Genomics, Chinese Academy of Sciences (GSA-Human: HRA004765) and are publicly accessible at https://ngdc.cncb.ac.cn/gsa-human.

### Sequencing analysis and data processing

The raw reads were filtered for high-quality clean reads using fast quality control and preprocessing tool (FASTP). Paired-end clean reads were merged into raw tags using fast length adjustment of short reads (FLASH), with a minimum overlap of 10 bp and a mismatch error rate of 2%.

The UPARSE pipeline (an OTU clustering algorithm) was employed to cluster the clean tags into OTUs with ≥97% similarity. Chimeric sequences were removed using the UCHIME (a chimera detection algorithm) algorithm. For each cluster, the representative sequence was defined as the tag with the highest abundance. Taxonomic annotations of the representative OTU sequences were performed using a naive Bayesian model with the ribosomal database project (RDP) classifier based on the SILVA (ribosomal RNA gene database) database, applying a confidence threshold of 0.8.

### Alpha/beta diversity and taxonomic analysis

Microbial diversity was assessed through OTU-based analysis. Alpha diversity was measured using the Sob, Chao, and ACE indices to evaluate within-sample species diversity. Beta diversity was assessed using principal coordinates analysis (PCoA) based on unweighted UniFrac and Bray–Curtis distances to compare microbial composition across samples. Venn diagrams were generated to highlight unique and shared OTUs between groups, while stacked bar plots were used to visualize differences in community composition. Species comparisons were made at both the phylum and genus levels.

### Identification of OTU-based markers and evaluation of the constructed models

Differential OTUs between healthy participants from Beijing and early LUAD patients across the three centers were identified using the Wilcoxon rank-sum test, with all identified OTUs included as candidate features. Participants from Beijing (70%) were randomly selected for the training cohort, with the remaining 30% assigned to the test cohort. Five commonly used ML algorithms—RF, support vector machine, decision tree, neural network, and elastic net—were applied for model development. For each model, twenty trials of ten-fold cross-validation were performed on the candidate OTUs in the training cohort using the train function in the caret R package, with parameters set to trainControl(method = “repeatedcv”, number = 10, repeats = 20). The importance of each OTU was recorded for each trial, and the mean importance across the 20 trials was used as the final importance value for each OTU. OTUs with an importance value ≥ the mean of all adopted OTUs plus one standard deviation were selected as the optimal biomarker set. This set was then used to train corresponding models in the training cohort, with the trained models applied to predict outcomes in the test and validation cohorts.

**Figure 1:**
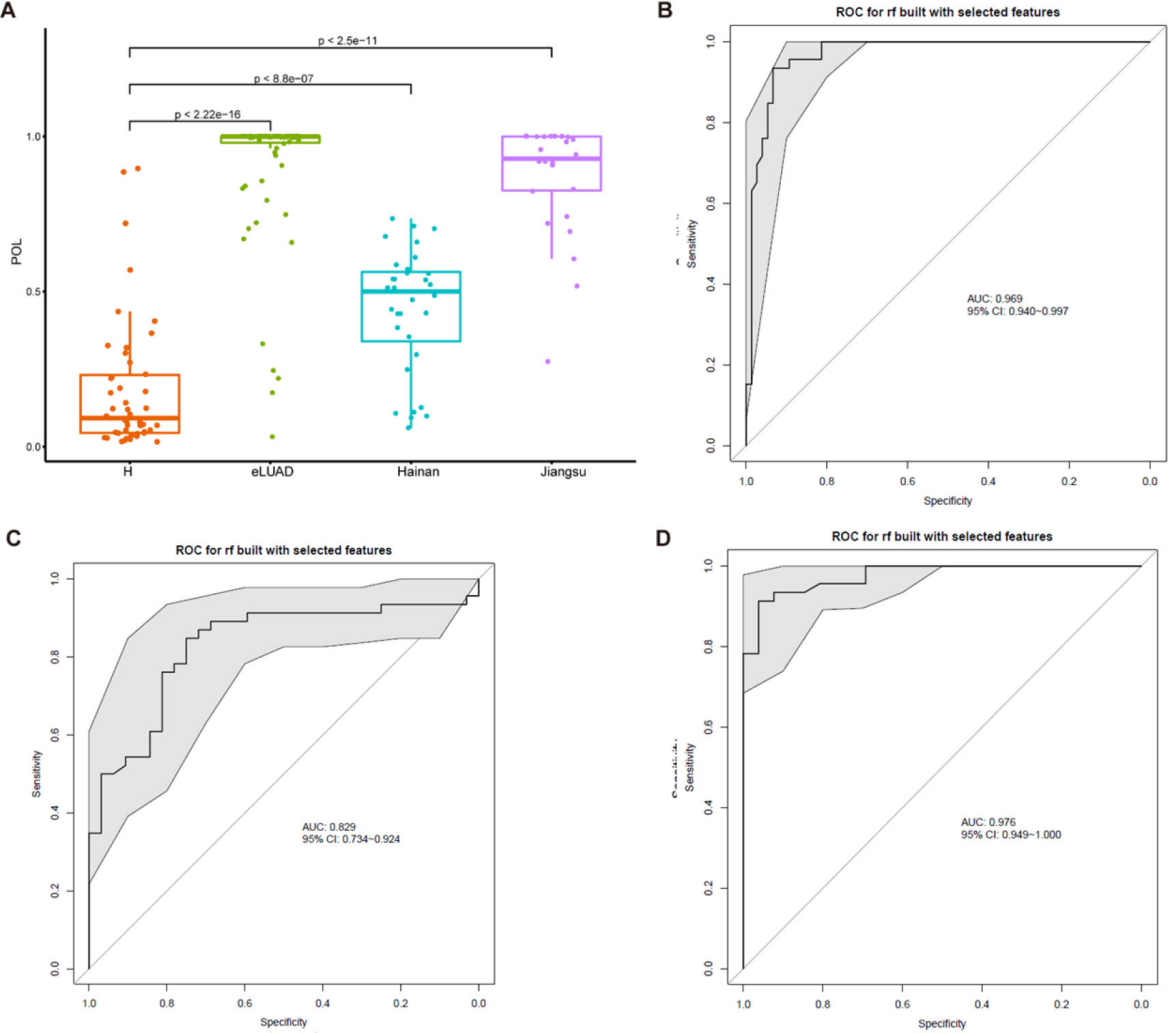
Validation OTUs-based markers for eLUAD by RF model. **(A)** The predicted POL was significantly increased in patients with eLUAD from the test cohort and the external cohorts versus the control participants (*P* < 2.2e-16, *P* < 8.8e-07, *P* < 2.5e-11, respectively). **(B)** ROC curve with an AUC value of 0.969 (95% CI: 0.940–0.997) in the test cohort from Beijing. **(C)** ROC curve with an AUC value of 0.829 (95% CI: 0.734–0.924) in the external cohort from Hainan. **(D)** ROC curve with an AUC value of 0.976 (95% CI: 0.949–1.000) in the external cohort from Jiangsu. eLUAD, Early lung adenocarcinoma; ROC, Receiver operating characteristic; AUC, Area under the curve; RF, Random forest; POL, probability of LUAD.

The probability of LUAD (POL) was derived from the RF model as the proportion of decision trees voting for each class. The POL index represented that the probabilities obtained using predict (model, newdata, type = “prob”) are fundamentally derived from the voting proportions across all decision trees in the forest. Specifically, for each sample to be predicted, every individual decision tree independently produces a hard classification (i.e., a vote for a specific class). The votes from all trees are then aggregated, and the number of votes for a given class is divided by the total number of trees, yielding the predicted probability that the sample belongs to that class. In **Figure 1A**, the y-axis represents the probability of each sample being classified into the ‘disease’ group within each subgroup. This metric intuitively reflects the model’s level of confidence in assigning a sample to the cancer group and is commonly used as a risk score when identifying biomarkers associated with cancer. Additionally, receiver operating characteristic (ROC) curves were generated to evaluate model performance.

### Statistical analysis

Statistical analyses were performed using R. Alpha diversity indices were calculated with QIIME (version 1.9.1). Principal coordinates analysis was conducted using the Vegan package (version 2.5.3), and the Adonis test was applied to assess statistical significance of the groupings. Venn analysis was performed using the VennDiagram package (version 1.6.16). Stacked bar plots were visualized with the ggplot2 package (version 2.2.1). Species comparisons were made using the Wilcoxon rank-sum test in the Vegan package (version 2.5.3). Statistical significance was set at P < 0.05.

## Results

### Characteristics of the training cohort

**Figure 2:**
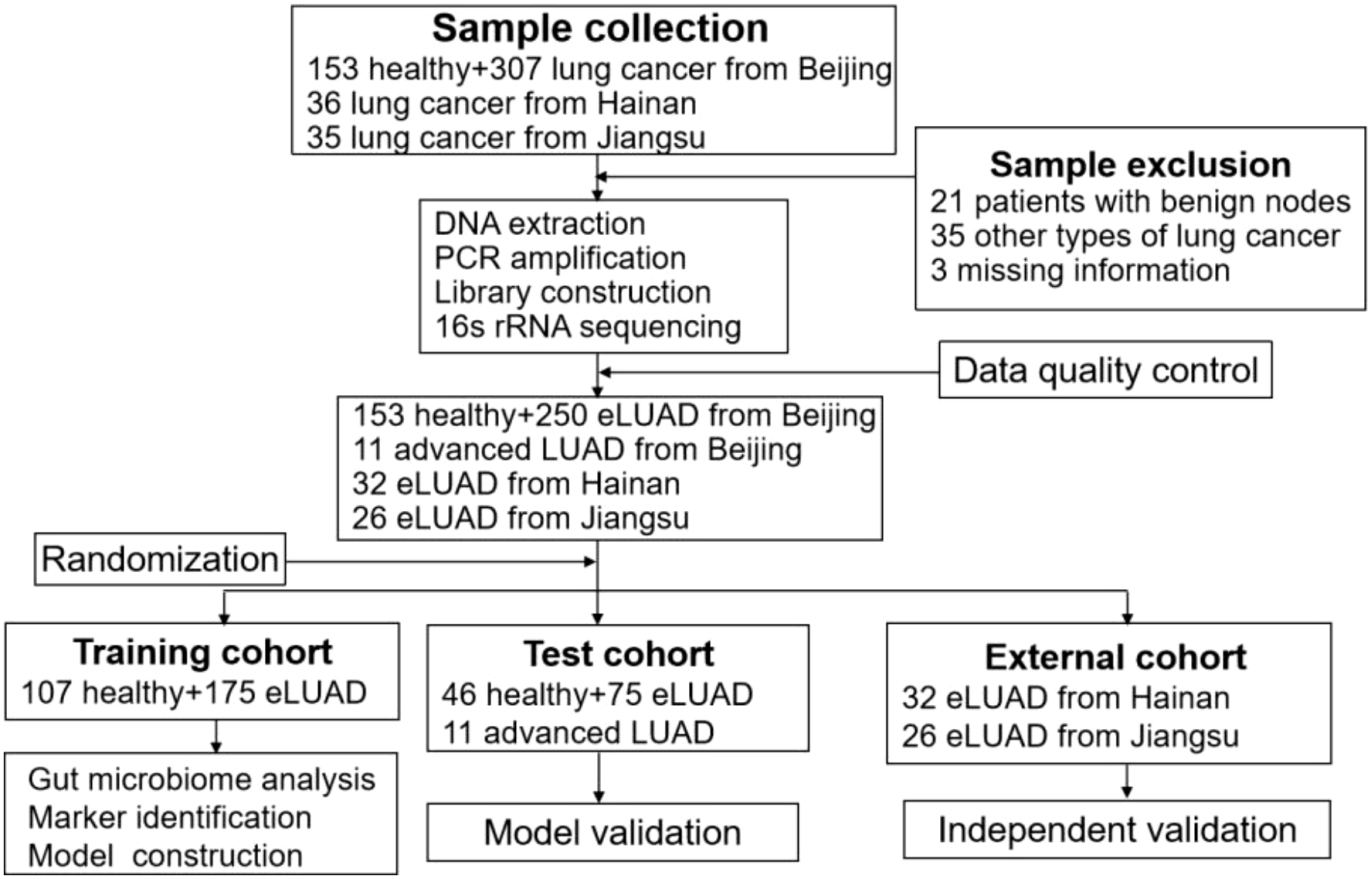
Study design and flow diagram. eLUAD, Early lung adenocarcinoma.

After a rigorous inclusion and exclusion process, 472 participants were enrolled, comprising 250 patients with early LUAD and 153 healthy controls from Beijing. These participants were randomly assigned to the training and test cohorts (**Figure 2**). The training cohort, which included 175 early LUAD patients and 107 healthy controls, was used to investigate gut microbial changes in early LUAD. Baseline data, including age, sex, body mass index, and smoking status, were collected for both cohorts. Pathological data, such as tumor stage and subtype, were recorded for LUAD patients. A summary of this information is presented in **Table 1**.

**Table 1:** Clinico-pathological characteristics of subjects in training cohort.

Variable names	eLUAD	Control
Age (year)	56.58 ± 11.01	51.17 ± 8.75
Gender		
Male	77 (44%)	63 (58.9%)
Female	98 (56%)	44 (41.1%)
BMI (kg/m^2^)	24.03 ± 3.14	25.52 ± 3.60
Smoking status (%)		
Never smoker	129	76
Ever smoker	46	31
Tumor stage		
0	2 (1.1%)	–
I	169 (96.6%)	–
II	4 (2.3%)	–
Tumor subtype		
PL	2 (1.1%)	–
MIA	20 (11.4%)	–
IA	153 (87.4%)	–

eLUAD, Early lung adenocarcinoma; BMI, Body mass index; PA, Preinvasive lesions; MIA, Minimally invasive adenocarcinoma; IA, Invasive adenocarcinoma.

### Gut microbial diversity alternation in early LUAD

**Figure 3:**
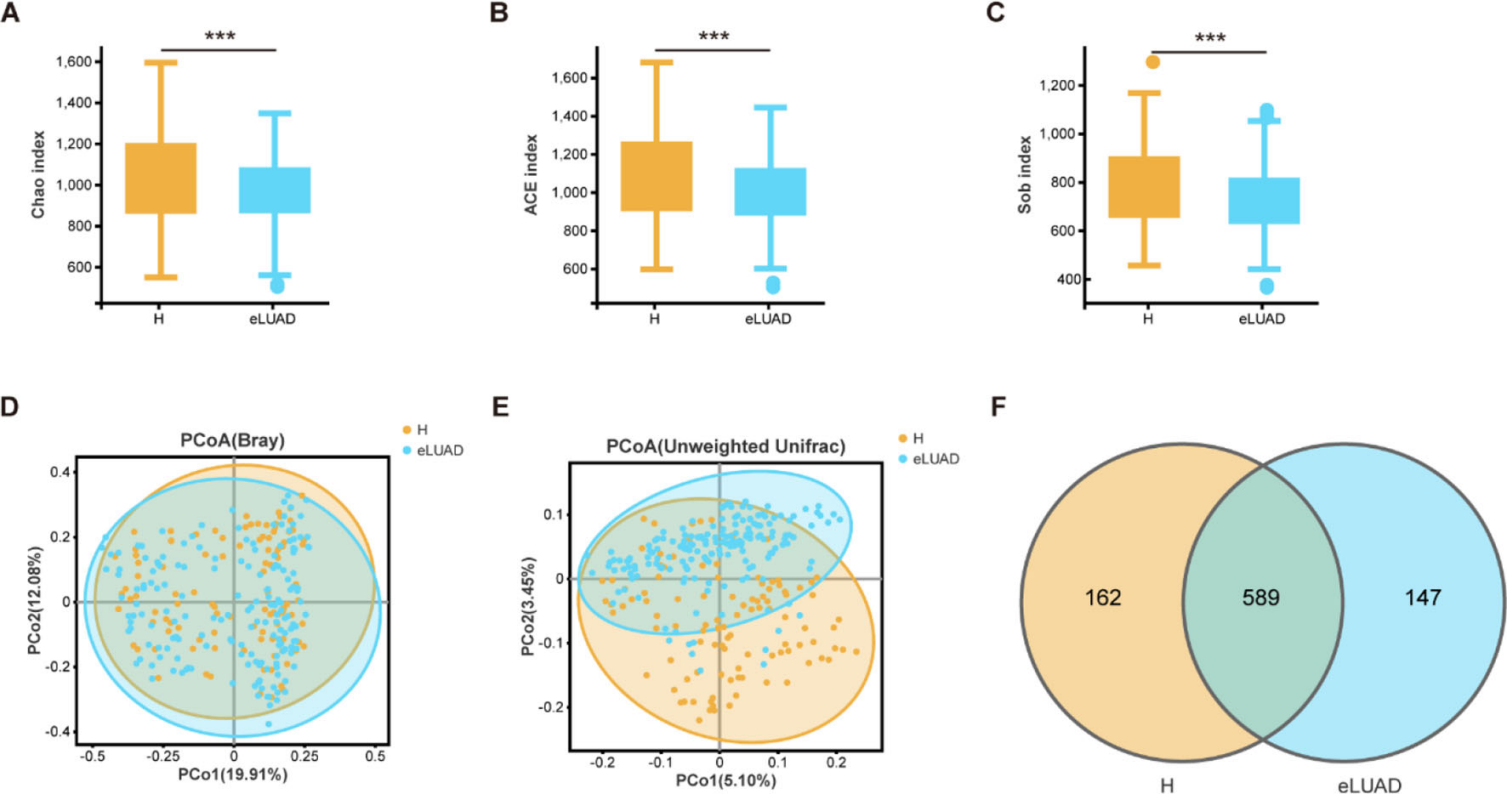
Gut microbial diversity alternation in patients with eLUAD (n = 175) compared to that in healthy subjects (n = 107). Alpha diversity was estimated using the Sob **(A)**, Chao index **(B)**, and ACE index **(C)**. **(D)**Beta diversity was evaluated using the PCoA of Bray–Curtis distance **(E)** and unweighted unifrac distance **(F)**. The common and unique OTUs between the healthy subjects and the patients with eLUAD are displayed in a Venn diagram. eLUAD, Early lung adenocarcinoma; PCoA, Principal coordinate analysis; OTUs, operational taxonomic units. “***” denotes statistical significance with P < 0.001.

Alpha diversity metrics were employed to assess microbial richness and evenness within samples. Compared to healthy controls, the Chao, ACE, and Sob indices were significantly reduced in patients with early LUAD (P = 0.001509, 0.000158, and 0.000065, respectively) (**Figures 3A–C**). Beta diversity, reflecting differences in microbial composition and distribution across samples, revealed significant distinctions between the two groups, as indicated by PCoA and the Adonis test (P = 0.006 and P = 0.001, respectively) (**Figures 3D, E**). Additionally, Venn analysis showed that the two groups shared 589 OTUs, with 162 OTUs unique to the healthy group and 147 OTUs exclusive to the early LUAD group (**Figure 3F**).

### Gut microbial features in early LUAD

**Figure 4:**
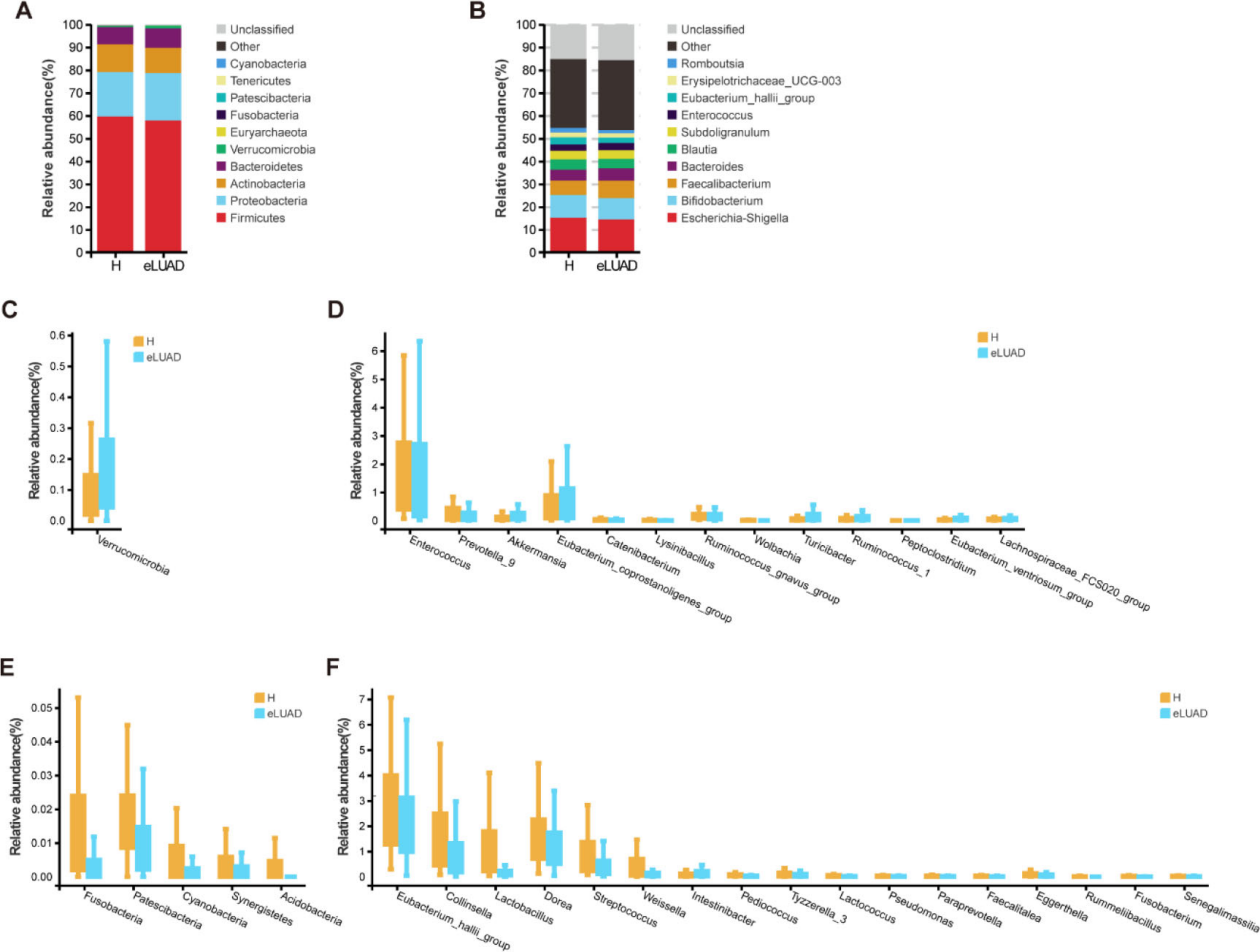
Differences in gut microbial community between patients with eLUAD and healthy subjects. Relative abundances of the predominant fecal microbiota between the two groups at the phylum **(A)** and genus levels **(B)**. Columns of different colours correspond to different species, while the length of the column represents their proportion. **(C)** The increased microbial community at the phylum level in patients with eLUAD versus control participants. **(D)** The predominantly increased microbial community at the genus level in patients with eLUAD versus control participants. **(E)** The decreased microbial community at the phylum level in patients with eLUAD versus control participants. **(F)** The predominantly decreased microbial community at the genus level in patients with eLUAD versus control participants. eLUAD, Early lung adenocarcinoma.

A stacked bar chart was generated for an intuitive comparison of sample abundance, highlighting the dominant species within each group and the expression trends between groups. The top 10 microbial taxa at the phylum and genus levels for both groups are shown in **Figures 4A, B**. Firmicutes, Proteobacteria, Actinobacteria, and Bacteroidetes were the most prevalent bacterial phyla in both groups, comprising 98.13% and 98.88% of the relative abundance in the early LUAD and healthy control groups, respectively. For taxa with a relative abundance greater than 0.1% in at least one sample, the Wilcoxon rank-sum test was applied to assess differences in median relative abundance between the groups. The phylum Verrucomicrobia was significantly more abundant in early LUAD patients compared to controls (**Figure 4C**), while Fusobacteria, Patescibacteria, Cyanobacteria, Synergistetes, and Acidobacteria were significantly decreased (**Figure 4E**). At the genus level, 13 genera, including *Enterococcus*, *Prevotella_9*, and *Akkermansia*, were more abundant in the early LUAD group (**Figure 4D**), whereas 17 genera, including *Eubacterium_hallii*_group, *Collinsella*, and *Lactobacillus*, were less abundant in the LUAD group compared to controls (**Figure 4F**).

### Identification of OTU-based markers for early LUAD by RF

**Figure 5:**
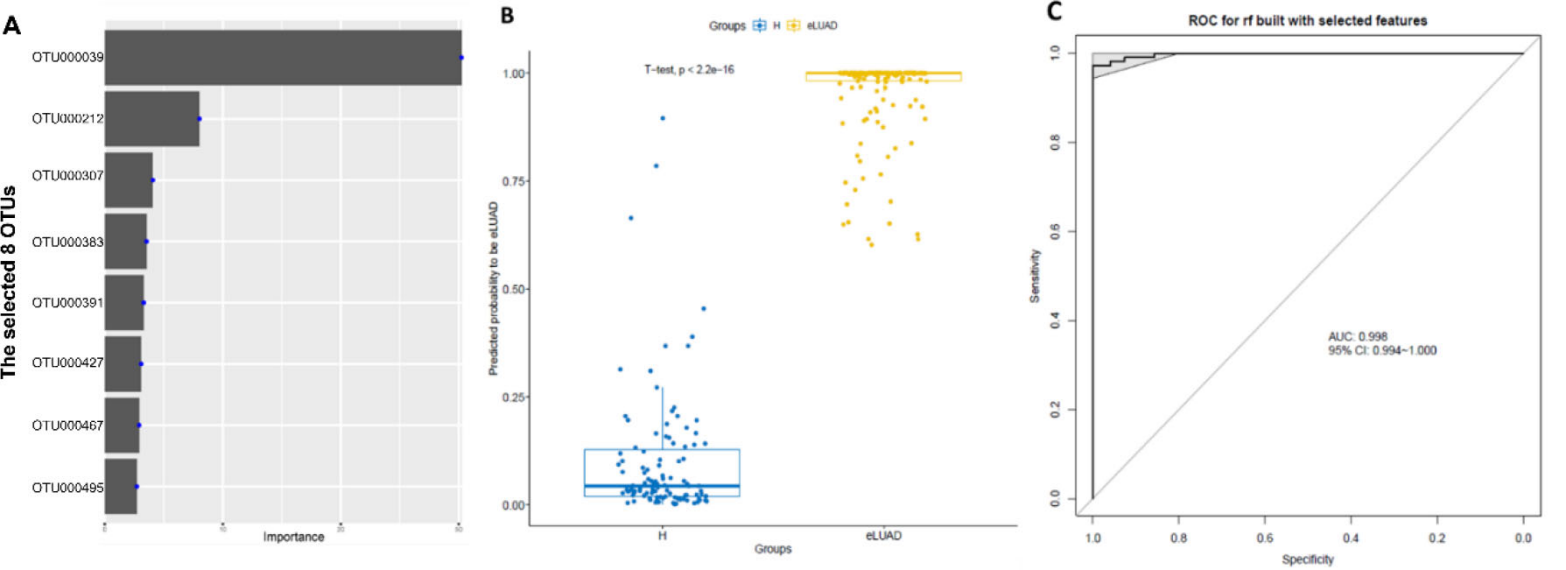
Identification of OTUs-based markers for eLUAD by RF model. **(A)** The selected OTUs were ranked by the importance value. **(B)** The predicted POL for eLUAD was significantly increased in patients with eLUAD versus the control participants (*P* < 2.2e-16). **(C)** ROC curve with an AUC value of 0.998 (95% CI: 0.994-1.000) by RF model in the training cohort. eLUAD, Early lung adenocarcinoma; ROC, Receiver operating characteristic; AUC, Area under the curve; RF, random forest; POL, probability of LUAD.

To identify a representative OTU combination capable of reliably distinguishing control participants from early LUAD patients, an RF model was trained. A total of 243 significantly differential OTUs between the two groups were included as candidate features for further analysis. In the training cohort, 20-fold cross-validation was performed, and 8 OTU-based markers were identified as the optimal set, ranked by their importance (**Figure 5A**). The POL index for each sample was calculated based on this optimal OTU set. The POL index was significantly higher in the early LUAD group compared to the healthy control group (**Figure 5B**). The model achieved an AUC of 0.998 (95% CI:0.994-1.000) in the training cohort (**Figure 5C**).

### Validation of OTUs-based markers for early LUAD

To validate the diagnostic performance for early LUAD, the trained model was applied to a test cohort comprising 75 early LUAD patients and 46 control participants. The predicted POL index was significantly elevated in early LUAD patients compared to controls (**Figure 1A**), with an AUC of 0.969 (95% CI: 0.940-0.997) (**Figure 1B**). Additionally, 32 and 26 early LUAD patients from Hainan and Jiangsu, respectively, were included as independent cohorts to further assess the diagnostic efficacy of the model. In both cohorts, the POL index was significantly higher in LUAD patients than in controls (**Figure 1A**), with the model achieving an AUC of 0.829 (95% CI: 0.734–0.924) in the Hainan cohort (**Figure 1C**) and an AUC of 0.976 (95% CI: 0.949–1.000) in the Jiangsu cohort (**Figure 1D**). These results demonstrated the widespread applicability and robustness of the diagnostic model.

### Application of OTUs-based markers for advanced LUAD

**Figure 6:**
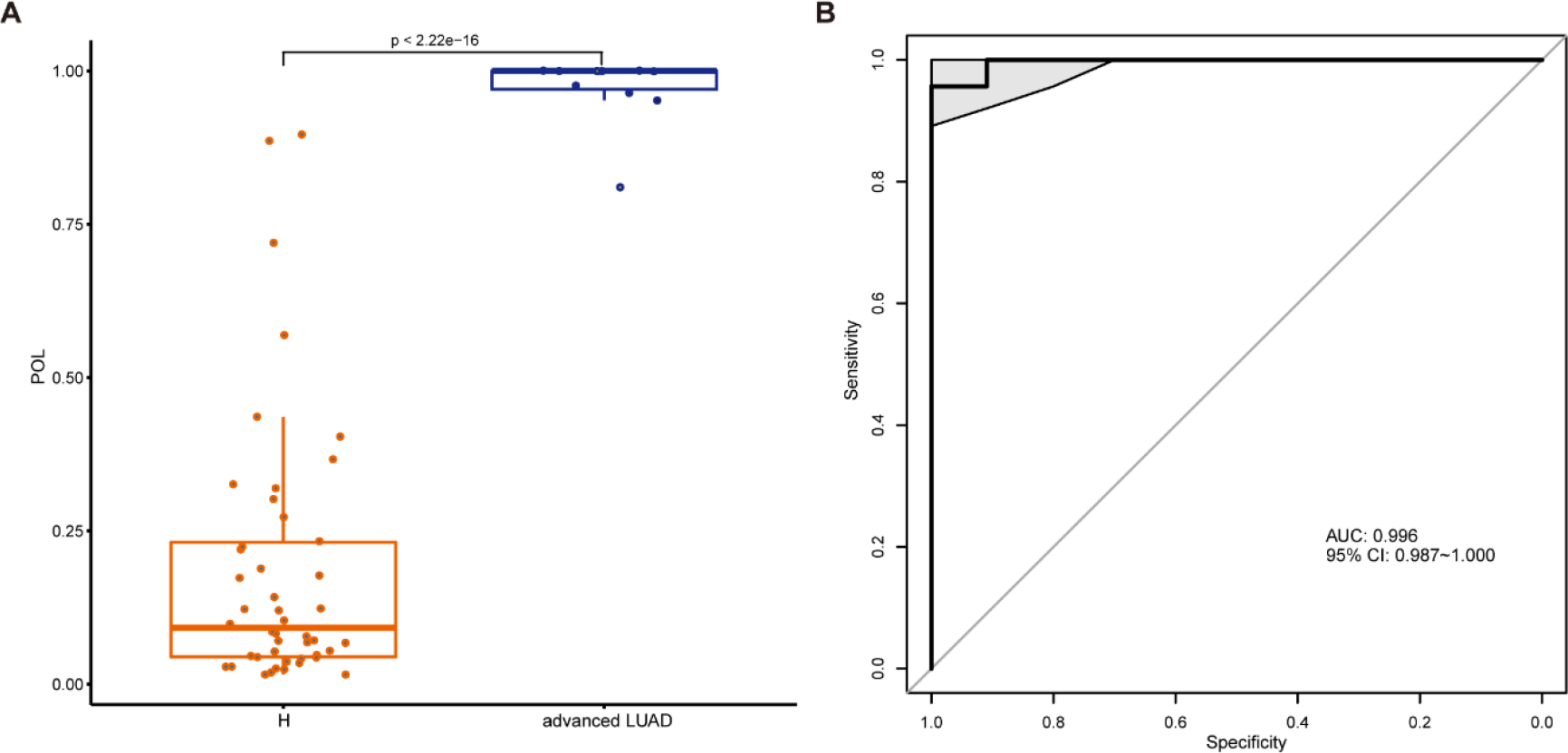
Application of OTUs-based biomarkers for advanced LUAD. **(A)** The comparison of the predicted POL between the 11 patients with advanced LUAD and the control participants. **(B)** ROC curve with an AUC value of 0.996 (95% CI: 0.987–1.000) between the advanced LUAD and control participants. ROC, Receiver operating characteristic; AUC, Area under the curve; POL, probability of LUAD.

To further evaluate the model’s diagnostic utility, 11 patients with advanced LUAD were recruited. The POL index in these patients was notably higher than in controls (**Figure 6A**), with the model achieving an AUC of 0.996 (95% CI: 0.987–1.000) (**Figure 6B**). These results indicate that the model not only provides strong diagnostic efficacy for early LUAD but also has potential for diagnosing advanced LUAD.

### Performance comparison with other models

To assess the advantages of the constructed model, four other commonly used ML algorithms—decision tree, elastic net, support vector machine, and neural network—were trained using the same candidate OTUs to distinguish early LUAD patients from controls. The performance metrics for all five models, including the RF model, are summarized in **Table 2**. In all cohorts, the RF model outperformed the other four models in terms of AUC. Additionally, the 8 OTU-based markers identified by the RF model were precise, making it a feasible approach for clinical implementation. Overall, the RF model demonstrated superior performance and greater diagnostic potential for early LUAD compared to the other models.

**Table 2:** Performance comparison with other models for diagnosing LUAD in all cohorts.

Models	DT	EN	RF	NN	SVM
The optimal OTU set	7	7	8	36	27
AUC (Training cohort)	0.913	0.567	0.969	0.980	0.671
AUC (Test cohort)	0.923	0.685	0.998	0.845	0.617
AUC (Hainan)	0.750	0.534	0.829	0.866	0.587
AUC (Jiangsu)	0.898	0.603	0.976	0.948	0.605
AUC (Advanced LUAD)	0.883	0.706	0.996	0.895	0.747

AUC, Area under the receiver operating characteristic curve; DT, Decision tree; EN, Elastic net; NN, Neural network; RF, Random forest; SVM, Support vector machine with radial kernel.

## Discussion

Our study includes 472 samples with 16S rRNA sequencing from multiple centers and incorporates independent validation cohorts, providing a more comprehensive and robust framework for evaluating the diagnostic potential of gut microbiome in LUAD. Previous studies investigating the gut microbiome in lung cancer were generally limited by relatively small sample sizes. For example, earlier 16S rRNA-based studies typically included fewer than 100 participants, such as cohorts of 42 patients with 34 validation cases (Zheng et al., [[NoYear]]). Although larger-scale studies have been reported, such as a recent analysis of 940 lung cancer cases published in Nature Communications, these investigations primarily focused on tumor tissue microbiome rather than gut microbiota and did not aim to develop non-invasive diagnostic models (McElderry et al., 2025). This study develops and validates OTU-based microbial markers for LUAD across multiple regionally distinct cohorts, and provides a comprehensive evaluation of their diagnostic performance through systematic comparison of multiple machine learning models. With advancements in high-throughput sequencing technology, ML models based on comprehensive gut microbiome features have gained significant attention for diagnosing numerous diseases, particularly cancers (Le Berre et al., 2020). For instance, Yu et al. discovered and cross-ethnically validated microbial biomarkers for CRC across participants from four countries (Yu et al., 2017). Ren et al. established a GM-based diagnostic model for HCC, validated across three different cohorts (Ren et al., 2019). Nagata et al. identified robust microbial signatures through metagenomic sequencing as accurate diagnostic biomarkers for PC in a multinational study (Nagata et al., 2022). A recent study utilizing 16S rRNA sequencing also highlighted the diagnostic potential of the GM for LC in a cohort from Shanghai (Kashyap et al., 2024). However, its limited sample size and absence of independent validation hinder the broader application and clinical translation of these findings.

In this study, 472 fecal samples with diverse characteristics, including sex, age, and body mass index, were collected from three distinct regions. Eight OTU markers were identified as the optimal set for early LUAD using the RF model, which achieved a significantly higher accuracy than previous research in the test cohort (AUC = 0.998 versus 0.764) (McElderry et al., 2025). Notably, the near-perfect performance observed in the training set (AUC = 0.998) should be interpreted with caution, as it may indicate potential overfitting. For that, we conducted 5 repetitions of 10-fold cross-validation. The resulting AUC values were consistently high (ranging from 0.916 to 1.000, with an average of approximately 0.97, **Supplementary Material 1**), accompanied by relatively narrow 95% confidence intervals. The stability of these high AUC values across different data splits and repeated experiments suggests that the model has strong generalization ability and is not overfitted to any specific training set. Then, we plotted the learning curve for the training set (**Supplementary Material 2**). As the training sample size increased, the mean accuracy rapidly improved and gradually plateaued, while the standard deviation (shaded area) decreased. Importantly, there was no progressively widening gap between the training and validation performance, further supporting the absence of overfitting. The cross-region reproducibility of the model reflects consistent microbial alterations in LUAD patients, even as gut microbiome can vary due to lifestyle, geography, and environmental factors (Gacesa et al., 2022). This aligns with findings from other recent cancer studies (Yu et al., 2017; Ren et al., 2019; Nagata et al., 2022). Additionally, the model demonstrated excellent diagnostic performance for advanced LUAD. Among the five common ML models evaluated, RF achieved the highest AUC value, confirming its superior predictive accuracy. These results support the potential of microbial marker-based models as a reliable and widely applicable screening tool for early LUAD.

Previous studies have highlighted the critical role of gut microbiome in the carcinogenesis of extra-gastrointestinal cancers. Recent advances in respiratory immunology and the emerging concept of the gut-lung axis have drawn attention to the link between intestinal microbiota and LC (Ge et al., 2021; Ye et al., 2025). Furthermore, animal model studies have demonstrated that commensal microbiota can inhibit LC development and extend survival by modulating lung immune responses (Li et al., 2012; Cai et al., 2025). In this study, a decrease in microbial diversity was observed in patients with LUAD compared to healthy controls, consistent with previous reports (Guo et al., 2023; Eslami et al., 2025). The microbial composition also differed significantly between the two groups, paralleling findings in tumor tissues, bronchoalveolar lavage fluid, and oral wash specimens from LC patients (Lee et al., 2016; Vogtmann et al., 2022; Li et al., 2024). These results provide a comprehensive assessment of gut microbiome alterations in LUAD patients, which may be linked to tumorigenesis and progression.

Distinct diseases are associated with unique microbial compositions (Nejman et al., 2020). *Akkermansia*, a mucin-degrading bacterium known for its immunoregulatory function through the production of short-chain fatty acids from mucin (Cani and de Vos, 2017; Patrizz et al., 2020), is inversely correlated with several diseases, including diabetes, inflammatory bowel disease, psoriatic arthritis, and HCC (Le Chatelier et al., 2013; Scher et al., 2015; Ren et al., 2019). In contrast, this study found an increased abundance of the phylum Verrucomicrobia and the genus *Akkermansia* in LUAD patients, aligning with findings in other cancers such as thyroid cancer, PC, and CRC (Shah et al., 2018; Ishaq et al., 2022; Kartal et al., 2022). Additionally, Verrucomicrobia has been shown to increase in neurological disorders, including stroke, multiple sclerosis, and Parkinson’s disease (Jangi et al., 2016; Stanley et al., 2018; Lin et al., 2019). These observations may be explained by the association of *Akkermansia* with interferon signaling, NF-kB signaling, and pro-inflammatory pathways, including the upregulation of genes involved in antigen presentation, B and T cell receptor signaling, and the complement cascade (Derrien et al., 2011; Ganesh et al., 2013). Notably, while no significant change was observed in the overall abundance of Firmicutes, many genera within this phylum, including *Lactobacillus*, *Streptococcus*, *Dorea*, and *Weissella*, were reduced in LUAD patients. As a well-known probiotic, *Lactobacillus* has been shown to enhance antitumor immunity by activating CD8+ T cells against oropharyngeal cancer through G protein-coupled receptor signaling (Wang et al., 2022). Similarly, in **Figure 5A**, feature importance analysis based on the random forest model revealed that OTU000039, classified as *Lactobacillus delbrueckii*, contributed most prominently to the discrimination between early LUAD and control samples (**Supplementary Material 3**). This finding suggests that microbial taxa within the genus *Lactobacillus* may play a role in the classification performance of the model. Biologically, *Lactobacillus delbrueckii* is a Gram-positive lactic acid bacterium widely recognized for its probiotic-associated properties and its role in maintaining microbial homeostasis. It has been extensively used in fermented foods and has demonstrated multiple health-related functions, including the production of bioactive metabolites and modulation of host physiological processes (Nami et al., 2026). Functionally, *Lactobacillus delbrueckii* contributes to host health by regulating inflammation, modulating gut microbiome composition, and maintaining epithelial barrier integrity (Lima de Jesus et al., 2024). In addition, previous studies have shown that this species exhibits immunomodulatory and anti-inflammatory properties, which may influence disease-related processes (Lima De Jesus et al., 2022). These findings suggest that *Lactobacillus delbrueckii* may participate in host–microbiome interactions and immune regulation. However, its specific role in lung adenocarcinoma remains unclear and requires further investigation.*Streptococcus* has also been found to be depleted in CRC patients, exerting a tumor-suppressive effect through the secretion of β-galactosidase (Li et al., 2021). Additionally, *Dorea*, a producer of acetate and lactate and a potential substrate for butyrate synthesis, is positively correlated with neoadjuvant chemotherapy responsiveness in breast cancer, likely mediated through the modulation of CD4+ T lymphocytes (Li et al., 2022). Similarly, several *Weissella* species have been investigated for their anticancer properties, as they suppress cancer cell growth and stimulate the immune system in immunocompromised patients (Ahmed et al., 2022). However, further research is required to fully elucidate the role of altered gut microbiome in LUAD pathogenesis.

A key strength of this study lies in its large-scale, multi-center cohort across diverse regions of China. Additionally, the use of five different ML models to train OTU-based data enabled the development of an optimal diagnostic classification. However, several limitations must be acknowledged. First, while 16S rRNA sequencing is widely used in microbiome research, it does not provide complete microbiota information, which could be obtained through metagenomic sequencing. Second, this was a cross-sectional study, and gut microbiome composition is known to be strongly influenced by environmental and lifestyle factors, particularly diet and smoking. Recent high-impact studies have demonstrated that dietary patterns play a central role in shaping gut microbial communities and contribute substantially to inter-individual variability (David et al., 2014; Asnicar et al., 2021). In addition, smoking has been shown to significantly alter gut microbiome composition and diversity, with partial reversibility following smoking cessation (Antinozzi et al., 2022; Leite et al., 2022). In our study, we just collected smoking status, detailed information on dietary habits and smoking time was not systematically collected, which represents a limitation. These unmeasured factors may introduce variability in the observed microbial profiles. However, the multi-center design and independent validation cohorts may partially mitigate this limitation by enhancing the robustness and generalizability of our findings. In future studies, incorporating comprehensive lifestyle data will be essential to further validate microbiome-based diagnostic models. At the same time, longitudinal studies accounting for such variables are necessary. Finally, it remains to be determined whether combining microbial biomarkers with existing clinical standard tests, such as CT scans and tumor biomarkers, could enhance diagnostic accuracy.

In conclusion, this study highlights characteristic changes in the gut microbiome of early LUAD patients, develops a predictive model based on microbial biomarkers, and rigorously validates its diagnostic efficacy using a large-scale multi-center cohort. These findings highlight the diagnostic potential of GM analysis as a targeted, non-invasive approach and contribute to our understanding of gut microbial alterations associated with LUAD. Such insights could play a pivotal role in the early diagnosis and effective treatment of LUAD in the future.

## Data Availability

The raw data supporting the conclusions of this article will be made available by the authors, without undue reservation. Raw sequencing data have been deposited in the Genome Sequence Archive at the National Genomics Data Center, China National Center for Bioinformation / Beijing Institute of Genomics, Chinese Academy of Sciences (GSA-Human: HRA004765) and are publicly accessible at https://ngdc.cncb.ac.cn/gsa-human.
