# Supplementary figures and images for: Identification and evaluation of gut microbiome as non-invasive biomarkers for early lung adenocarcinoma from a multi-center study
Source: Front Cell Infect Microbiol. 2026 May 14;16:1813261. doi: 10.3389/fcimb.2026.1813261 (PMC13216471; doi:10.3389/fcimb.2026.1813261)

# Learning Curve

Solid line: Mean Accuracy; Shaded area: Standard Deviation (Stability)

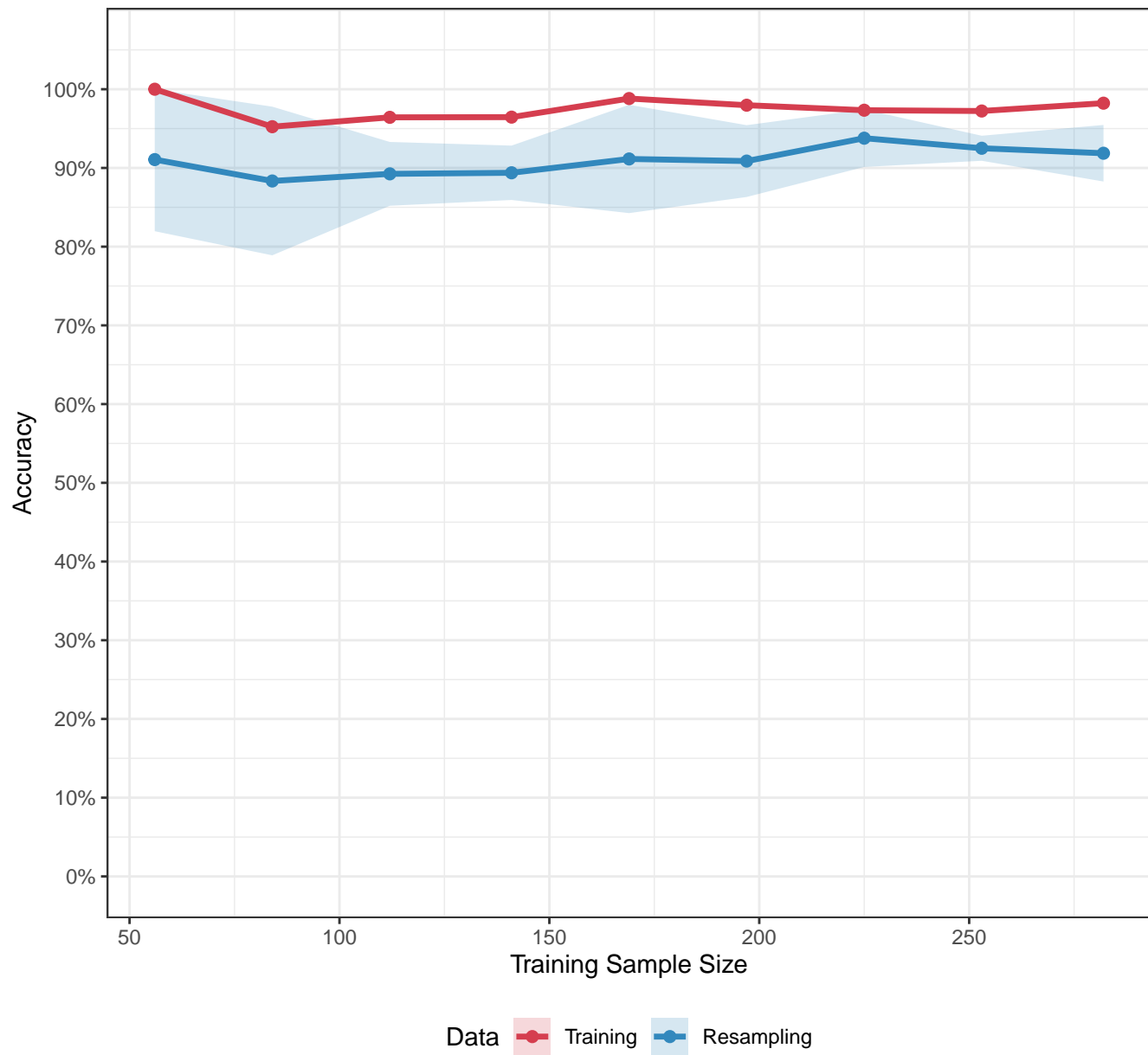

Supplement: Supplementary file 3 [file Image1.pdf]
